# Supplementary material for: GradNav: Accelerated Exploration of Potential Energy Surfaces with Gradient-Based Navigation
Source: J Chem Theory Comput. 2024 May 10;20(10):4088–98. doi: 10.1021/acs.jctc.4c00316 (PMC11137815; doi:10.1021/acs.jctc.4c00316)
Supplement: Supplementary file 1 — ct4c00316_si_001.pdf [file ct4c00316_si_001.pdf]

Supporting Information:
GradNav: Accelerated Exploration of Potential Energy Surfaces with Gradient-Based Navigation

Janghoon Ock,[†] Parisa Mollaei,[‡] and Amir Barati Farimani[*,‡]

†*Department of Chemical Engineering, Carnegie Mellon University, 5000 Forbes Street, Pittsburgh, PA 15213, USA*

‡*Department of Mechanical Engineering, Carnegie Mellon University, 5000 Forbes Street, Pittsburgh, PA 15213, USA*

E-mail: barati@cmu.edu

Contents

S1 Outer Loop Trajectory **S2**

S2 Fs-Peptide Protein **S3**

S3 Update of Initial Points in Fs-Peptide Trajectories **S4**

References **S4**

S1 Outer Loop Trajectory

Each trajectory generated during an iteration of the outer loop simulation can be considered a reliable segment of simulation, assuming the frame duration is appropriately set. The left panels of Figure S1 display trajectories exclusively obtained from outer loop runs. Upon examining the right panels in Figure 2, one observes trajectories more densely localized within potential wells. The majority of the dispersed points located in the high energy area of the right panels in Figure 2 correspond to the initial positions for each run of the inner loop, as depicted in the right panels of Figure S1.

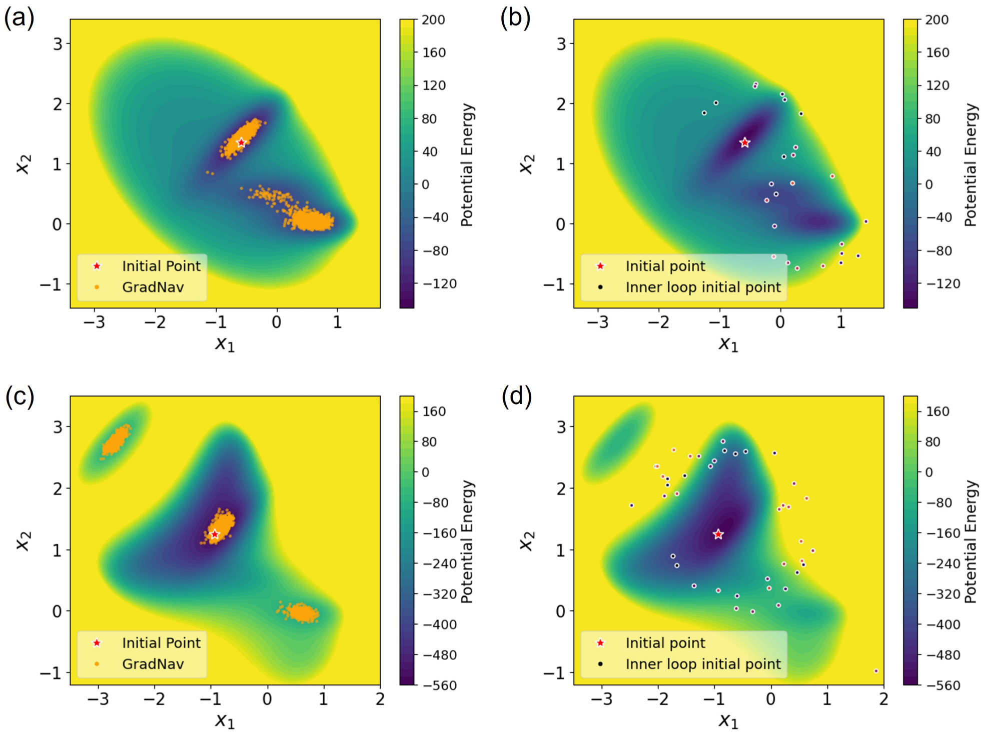

Figure S1: Trajectories from outer loop Simulations. The upper panels **a** and **b** showcase results from simulations using the Müller potential, while the lower panels **c** and **d** depict outcomes from the modified Müller potential.

S2 Fs-Peptide Protein

In one of our studies,[S1] we investigated the dynamics and conformational states of individual amino acids within proteins using a combination of molecular dynamics (MD) simulations and machine learning (ML) techniques. Specifically, we focused on identifying residues that switch between two distinct angular states, classifying them as either stable switch (ALA9 in Figure 6) or unstable switch (ARG20 in Figure 6) residues, and evaluating their contribution to the overall properties of proteins. For instance, in the Fs peptide protein, we found that the Root Mean Square Deviation (RMSD) feature, indicative of protein folding, strongly correlates with the dynamics of ALA9 amino acid. It suggests that the ALA9's dynamics may contribute to the folding process of the protein. However, the ARG20 jumps between two distinct angular states regardless of the conformational states of the protein. As Figure 6 shows, the ALA9 residue is stable in a single angular state when the protein is unfolded (Figure 6b) and suddenly switches to another state once the RMSD increases (Figure 6c). While ARG20 transitions between the two angular states irrespective of the protein's conformational states.

S3 Update of Initial Points in Fs-Peptide Trajectories

We assess the implementation of GradNav in Fs-Peptide molecular dynamics simulations through a pseudo molecular dynamics approach. In this method, a tentative initial point is proposed using the update rule (Eqn 1). Subsequently, the actual new starting point is chosen from among those points that lie within a specified cutoff radius (0.02) from the proposed initial point. Given that the dataset includes 280,000 trajectory frames, thoroughly covering the molecular space with diverse topologies, we consistently identified multiple points within the cutoff radius, ensuring only a slight variation between the proposed new starting point and the actual new starting point, as demonstrated in Figure S2.

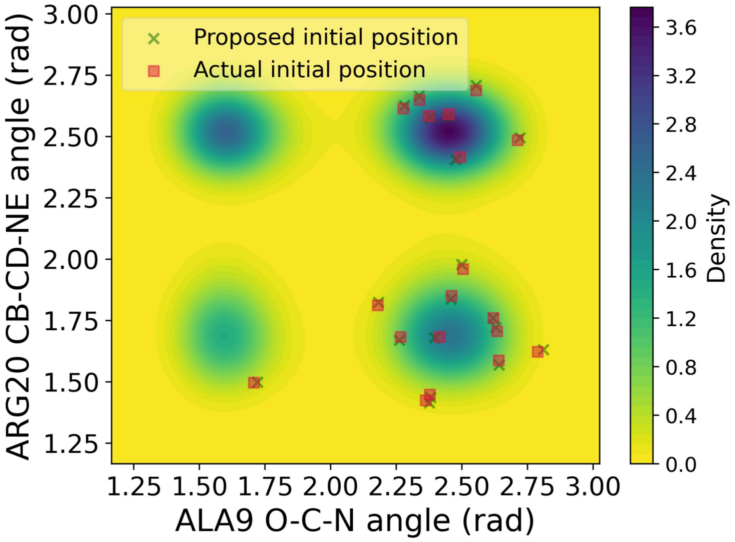

Figure S2: Proposed vs. actual initial points. The proposed initial points are updated using the GradNav algorithm's update rule, while the actual initial points are selected from the trajectory dataset based on a cutoff radius of 0.02.

References

(S1) Mollaei, P.; Barati Farimani, A. Unveiling Switching Function of Amino Acids in Proteins Using a Machine Learning Approach. *Journal of Chemical Theory and Computation* **2023**, *19*, 8472–8480.
